# Strength Assessment Under Dual Task Conditions in Women with Fibromyalgia: A Test–Retest Reliability Study

**DOI:** 10.3390/ijerph16244971

**Published:** 2019-12-06

**Authors:** Juan Luis Leon-Llamas, Santos Villafaina, Alvaro Murillo-Garcia, Daniel Collado-Mateo, Francisco Javier Domínguez-Muñoz, Jesús Sánchez-Gómez, Narcis Gusi

**Affiliations:** 1 Faculty of Sport Sciences, University of Extremadura, 10003 Cáceres, Spain; leonllamas@unex.es (J.L.L.-L.); jesanchezg@unex.es (J.S.-G.); ngusi@unex.es (N.G.); 2 Centre for Sport Studies, Rey Juan Carlos University, 28943 Fuenlabrada, Spain; danicolladom@gmail.com

**Keywords:** dual-task, chronic pain, activities of daily living, strength

## Abstract

The present study aimed to: (1) analyze the test–retest reliability of the 30 s chair stand test and the 30 s arm curl test under dual-task conditions; (2) analyze the test–retest reliability of a new variable which assesses the total performance (cognitive + physical) in both tests. A total of 37 women with fibromyalgia participated in the study. Participants completed the 30 s arm curl test and 30 s chair stand test in both simple and dual-task conditions. These tests were repeated after seven days. In the 30 s chair stand dual-task test the reliability was low to good whereas that of the total performance variable was low to moderate. The reliability in both the 30 s arm curl dual-task test and the total performance variable were good to moderate. Both the 30 s chair stand test and 30 s arm curl test under dual-task conditions and the total performance variables had good test–retest reliability. However, it is necessary to consider the fluctuations of the intraclass correlation coefficient (ICC).

## 1. Introduction

Fibromyalgia (FM) is defined as a chronic disease characterized by persistent, diffuse, and widespread pain associated with several symptoms. Amongst others, these symptoms include non-recovery sleep, anxiety, depression, stiffness, fatigue, cognitive problems, and mobility or balance problems [1]. Therefore, most people who suffer from FM tend to experience a reduced quality of life [2,3,4,5], as well as a series of difficulties related to activities of daily living [6,7,8].

In Europe, the prevalence of this disease is somewhere between 2.9% and 4.7% of the general population [9], showing higher incidence in women aged 40–59 years [2,10]. In economic terms, it is estimated that FM represents twelve billion euros annually for a population of 80 million inhabitants [11]. In Spain, FM represents a total average of 9982 euros per patient, of which 32.5% is attributable to health care costs and 67.5% to indirect costs [12]. Physical exercise is shown as an interesting tool that improves fitness and wellness in this population [13], therefore reducing health economic costs. Several studies have analyzed the importance of fitness in people with FM with regards to pain [14], quality of life [15], psychological disorders [16], and fear of falling [17].

Chronic pain significantly affects function and quality of life for patients with FM. This leads to a reduction in lower limb strength, agility, and balance [18]. In this regard, strength takes a particular interest because it determines the ability to carry out work, as well as activities of daily living [19,20]. Moreover, previous studies have demonstrated reduced muscle performance in patients with FM [21,22].

In an adult population, the most commonly used tests in the assessment of strength are the 30 s chair stand test and the 30 s arm curl test [23]. The first involves repeated sitting down and rising from a chair. The second involves flexing and extending the elbow throughout its range of motion, with an external weight of 2.5 kg [23]. In women with FM, these tests have been used to establish classifications based on the presence or absence of the disease as well as the severity of the symptoms [24,25].

Traditionally, physical fitness tests have been carried out by only involving the participant in a physical task. However, it would be interesting to approach these tests through activities of daily living. Activities of daily living usually involve two or more tasks simultaneously [26]. The dual-task paradigm proposes the accomplishment of two tasks simultaneously—a first cognitive task directs or focuses the attention of the participant towards an external source of attention (i.e., performing a mathematical operation) whilst performing a second motor task (i.e., climbing stairs). The different task combinations are motor–motor, cognitive–cognitive, and motor–cognitive. Previous studies have analyzed the influence of adding a cognitive task when performing physical fitness tests in women with FM [27,28,29], but none of them have reported test–retest reliability. A test is considered reliable when, on two or more occasions under the same conditions, a subject obtains similar results [30]. Therefore, the aim of the present study was to analyze the test–retest reliability of the 30 s chair stand test and 30 s arm curl test under dual-task conditions. This would allow us to determine whether the application of this kind of test closer to typical conditions of activities of daily living, could be reliable in women with FM. The secondary aim was to analyze the test–retest reliability of the new variable created, which assesses the total performance (cognitive + physical). Accordingly, the hypotheses of the present study were: (1) that the 30 s chair stand test and the 30 s arm curl test under dual-task conditions are reliable in women with FM; (2) that the variable which assesses the total performance (cognitive + physical) is reliable in both tests.

## 2. Materials and Methods

### 2.1. Participants

A total of 37 women from a local FM association participated in this study. This sample size with two observations per subject achieves 91% power to detect an intraclass correlation of 0.90 under the alternative hypothesis, when the intraclass correlation under the null hypothesis is 0.75 using an F-test with a significance level of 0.05 [31,32]. The PASS software for performing power and sample size calculations (version 11.0; PASS; Kaysville, Utah) was employed.

The main characteristics are shown in Table 1. The following inclusion criteria were established for this study: (a) female between 30 and 75 years old; (b) diagnosed with FM by a rheumatologist according to the 2010 criteria established by the American College of Rheumatology [1]; (c) able to communicate effectively with the study staff; and (d) understood and signed informed consent in accordance with the updated Declaration of Helsinki. Participants were excluded if they: (a) were pregnant; (b) could not sit down and get up from a chair without help; or (c) had an arm injury that prevented flexion and extension of the elbow. This study obtained the agreement of the Biomedical Ethics Committee of the University of Extremadura (Spain) (62/2017).

### 2.2. Procedure

First, anthropometric measurements of the participants were taken to calculate the body mass index (BMI), as well as age and years with FM. Subsequently, participants completed the Spanish version of the Fibromyalgia Impact Questionnaire (FIQ), which evaluates the impact of symptoms of the disease from 0 to 100, indicating the minimum to maximum impact respectively [3,33,34]. Finally, two physical fitness tests were performed: (1) the 30 s arm curl test and (2) the 30 s chair stand test. Tests were performed in two conditions—simple and dual-task. The dual-task conditions consisted of subtracting two by two from a random number greater than 100. The cognitive task was completed continuously throughout each 30 s physical performance. The order of simple and dual-task was randomized in the test and retest. In the same sense, in order to avoid a learning effect, the evaluations were repeated after seven days [35].

### 2.3. 30 s Arm Curl Test

The 30 s arm curl test was performed according to the recommendations of Boneth Collantes et al. [36]. Participants sat in a chair with a straight back supported by a backrest, with the trunk perpendicular to the floor and the feet placed fully on the floor. A weight of 2.5 kg was given to the participants. They held the weight in the dominant hand with the wrist in a neutral position and the elbow in extension. To complete the exercise, the participant was asked to flex the elbow and then return to full extension with the wrist in a neutral position. This cycle was repeated as many times as possible in 30 s. Before starting the test, a member of the study staff demonstrated the execution and allowed it to be practiced twice by the participant, to ensure it was correctly performed and to serve as a familiarization for the test. The test began to the command of “ready, go”. The number of flexions and extensions of the elbow joint that were performed in 30 s were recorded so long as the participant performed a correct movement.

### 2.4. 30 s Chair Stand Test

The 30 s chair stand test was performed according to the recommendations of Boneth Collantes et al. [36]. Participants started by sitting in a chair with their arms crossed and fixed at chest level, placing their hands on their shoulders. They stood up from the sitting position to full knee extension, and then returned to the initial position until the back was supported by the backrest. This cycle was repeated as many times as possible in 30 s. The time and the repetitions were measured by using the free software Chrono-jump (Chronojump-BoscoSystem, Barcelona, Spain) with the open hardware Chronopic [37]. Before starting the test, a member of the study staff demonstrated the execution and allowed it to be practiced twice by the participant to ensure it was correctly performed and to serve as familiarization for the test. The test began to the command of “ready, go”. The number of times that the participant sat in the chair in a period of 30 s was recorded, so long as the participant performed a correct movement.

### 2.5. Statistical Analysis

The SPSS statistical package (version 24.0; IBM Corp., Armonk, NY, USA) was employed. Parametric and non-parametric tests were conducted based on the results obtained in the Shapiro–Wilk test. The differences between test and retest were evaluated using the independent *t*-test or Wilcoxon signed-rank test when appropriate.

Reliability was estimated and their 95% confidence intervals are reported using recommendations by Weir [38]. The intraclass correlation coefficient (ICC) two-way random effects, consistency, and single measurement [39] were chosen. In the same way, recommendations by Koo et al. [39] were used for the interpretation of the ICC estimates and their 95% confidence intervals. Therefore, the 95% confidence intervals of the ICC estimates should be used to interpret the level of reliability. Values lower than 0.5 indicate poor reliability, values between 0.5 and 0.75 indicate moderate reliability, values between 0.75 and 0.9 indicate good reliability, and values greater than 0.9 indicate excellent reliability. Absolute reliability was established by calculating the standard error of measurement (SEM) following this formula:

(1) $$\mathrm{SEM}=\mathrm{SD}\;\sqrt{1-\mathrm{ICC}},$$

where *SD* is the mean standard deviation of the two repetitions (test–retest). The smallest real difference (SRD) was calculated according to the formula:

(2) $$\mathrm{SRD}=1.96\times \mathrm{SEM}\times \sqrt{2}.$$

Both SEM and SRD were expressed in percentages in order to facilitate comparison with previous and future studies.

Total performance (TP) was calculated taking into account the physical performance (number of repetitions) and the cognitive performance (number of successes and errors in the cognitive test) through the formula:

(3) TP=number of repetitions test+(number of successes−number of errors).

## 3. Results

Table 1 shows the main characteristics of the participants. The mean age was 54.76 (8.64) years, with a BMI of 28.30 (3.43) and a FIQ score of 53.61 (19.88).

Table 2 shows the reliability parameters obtained for each of the tests performed. Following the recommendations of Koo et al. [39], the lower and upper bound of the 95% confidence interval of the ICC estimate was used to interpret the level of reliability. Therefore, a moderate to excellent reliability index (95% CI: 0.726 to 0.921) was observed for the number of repetitions in the 30 s chair stand test during the single-task conditions. A low to good reliability index (95% CI: 0.399 to 0.799) was observed for the number of repetitions in the 30 s chair stand test during the dual-task conditions. A low to moderate reliability index (95% CI: 0.151 to 0.678) was observed for the variable total performance (TP) in the 30 s chair stand test during the dual-task conditions. Furthermore, a moderate to excellent reliability index (95% CI: 0.718 to 0.918) was observed for the number of repetitions in the 30 s arm curl test during the single-task conditions. A good to moderate reliability index (95% CI: 0.650 to 0.895) was observed for the number of repetitions in the 30 s arm curl test during the dual-task conditions. Finally, a good to moderate reliability index (0.564 to 0.867) was observed for the variable TP in the 30 s arm curl test during the dual-task conditions.

## 4. Discussion

The main objective of this study was to establish the test–retest reliability in the 30 s arm curl and 30 s chair stand tests during dual-task conditions. Results indicate that both tests can be considered reliable, as can the total performance (TP) variable calculated for both tests. However, it is necessary to take into account the ICC fluctuation range. This is the first study to analyze the test–retest reliability of the 30 s arm curl and 30 s chair stand tests under dual-task conditions in women with fibromyalgia (FM). The results confirm the idea of applying dual-task conditions as a reliable and ecological tool to approach physical fitness evaluation in real-life conditions.

Regarding absolute reliability, the SEM results can be considered high in the dual-task conditions. There was an 86.18% probability (with a 95% CI) that a repeated measure in the 30 s chair stand dual test differed 1.42 repetitions from the initial score. In the same way, there was an 87.23% probability that a repeated measure in the 30 s arm curl test differed 1.85 repetitions from the initial score.

The results obtained in the SRD indicate that improvements in the dual-task conditions higher than 3.93 in the 30 s chair stand test and 5.12 in the 30 s arm curl test would indicate a change in the measure that is not due to the error of the measure or the variability of the subject [40,41]. This is pertinent to helping healthcare professionals and researchers identify relevant functional changes in women with FM.

In order to quantify the TP of the tests, a specific variable, “total performance” was developed. This variable combines the number of successes, errors, and repetitions performed by the participants in the time test. Our method gives the same importance to the motor and cognitive parts since these components are crucial in activities of daily living and could provide us with relevant information when evaluating women with FM. Several studies have quantified the physical performance in these tests and this population [27,29]. Other studies have also measured test–retest reliability, but only in simple-task conditions [35,37]. Although previous research created a variable that included both physical and cognitive performance, the reliability of this method was not evaluated [42]. In this regard, this is the first study that demonstrates a low to moderate reliability in the 30 s chair stand test and a good to moderate reliability in the 30 s arm curl test, under dual-task conditions, in a variable that includes both physical and cognitive performance. Therefore, future studies that assess physical fitness under the dual-task paradigm can use this method to evaluate the total performance.

Previous studies have analyzed the impact of pain on the performance of activities of daily living in chronic pain populations such as rheumatoid arthritis [43] and FM [44]. Similarly, increases in pain and fatigue have been observed after dual-task conditions in patients with FM versus healthy subjects [45], concluding that pain can exert some negative influence on the development of activities of daily living. In this way, we can observe the impact that pain can have in patients with FM and the “conflict of objectives” [46] that can be generated in their daily life (e.g. to avoid personal pain or to meet with friends). In this regard, previous studies confirmed that pain is able to affect brain areas involved in attention processes, cognitive processing, memory and nociception [47,48], which could reduce the performance of activities of daily living. A previous study evaluated the relationship between fear of pain and physical performance under dual-task conditions [49]. The authors showed that baseline pain determines the level of physical activity that the person was willing to perform regardless of the pain that was generated in the test or the instructions given to reduce the pain. In this sense, our research did not specifically record the degree of pain that participants felt during the physical fitness tests, as in the study by Gier et al. [49]. However, future research is needed to clarify the effects of pain on dual-tasks and how pain can modulate the physical or cognitive performance of patients with FM, since it is a common feature in their daily life.

Given that activities of daily living simultaneously involve the performance of physical and cognitive tasks, the clinical relevance of the current study is due to the potential advantages of dual-task assessment by health professionals and researchers. This study also reports the number of repetitions that should be considered as a real change when these physical tests are performed along with the cognitive task, and can serve as reference values for the interpretation of results in future research. In the same way, the variable created to know the physical and cognitive performance of the participants can be used in both FM and other populations.

The present study has some limitations. First, the relatively small sample size that was only composed of women, means that the findings cannot be generalized to men. Secondly, data from cognitive tasks, under single-task conditions, were not included in the analysis, so the interference caused by the physical test on the cognitive test was not evaluated. The third limitation could be related to the potential learning from the test to the retest, which is a common limitation in test–retest reliability studies. Finally, the sample age ranged between 30 and 75 years old, which could have influenced the physical and cognitive performance.

## 5. Conclusions

Both the 30 s chair stand test and the 30 s arm curl test under dual-task conditions obtained good levels of test–retest reliability. Therefore, these tests could be considered as reliable tools for the evaluation of strength in women with fibromyalgia. In the same way, the total performance variable presented good levels of reliability. Future research with larger samples is required in order to generalize these results.

## Figures and Tables

**Table 1:** Descriptive characteristics of the participants.

Participants	Mean (SD)
Sample size	37
Age (years)	54.76 (8.64)
Years with fibromyalgia	12.73 (6.75)
BMI (kg/m^2^)	28.30 (3.43)
FIQ-100	53.61 (19.88)

FIQ: Fibromyalgia Impact Questionnaire; SD: standard deviation.

**Table 2:** Reliability of the 30 s chair stand test and arm curl test under single and dual-task conditions (*n* = 37).

Variable		Test	Retest	*p*-Value	Distribution Value	ICC (95% CI)	SEM	%SEM	SRD	%SRD
**30 s Chair Stand Test**	M (SD)	11.14 (2.65)	11.73 (2.36)	0.011	0.060	0.85 (0.726–0.921)	0.97	8.48	2.69	23.52
	Md (IQR)	11.00 (3)	12.00 (3)							
**30 s Chair Stand dual Test**	M (SD)	9.97 (2.45)	10.54 (2.28)	0.072	0.117	0.64 (0.399–0.799)	1.42	13.82	3.93	38.30
	Md (IQR)	10.00 (4)	10.00 (3)							
**TP 30 s Chair Stand Test**	M (SD)	22.70 (7.84)	23.16 (7.97)	0.776	0.782	0.62 (0.151–0.678)	4.85	21.14	13.44	58.60
	Md (IQR)	24.00 (12.00)	23.00 (11.50)							
**30 s Arm Curl Test**	M (SD)	16.64 (4.41)	16.24 (4.71)	0.471	0.005	0.85 (0.718–0.918)	1.79	10.88	4.96	30.17
	Md (IQR)	16.00 (6)	16.00 (5)							
**30 s Arm Curl Dual Test**	M (SD)	14.35 (4.34)	14.59 (4.03)	0.618	0.312	0.81 (0.650–0.895)	1.85	12.77	5.12	35.40
	Md (IQR)	14.00 (5)	14.00 (5)							
**TP 30 s Arm Curl Test**	M (SD)	28.31 (9.60)	29.95 (7.76)	0.070	0.024	0.86 (0.564–0.867)	3.26	11.19	9.03	31.01
	Md (IQR)	28.00 (10.25)	30.00 (8.50)							

M: mean; SD: standard deviation; Md: median; IQR: interquartile range; ICC: intraclass correlation coefficient; SEM: standard error of measurement; SRD: smallest real difference; TP: total performance. Paired *t*-test or Wilcoxon tests were conducted depending on the distribution (variables with a *p*-value lower than 0.05 in the Shapiro–Wilk test were considered for a non-parametric analysis).
